# Progress in Medicine

**Published:** 1896-06-27

**Authors:** 


### Progress in Medicine.

### PERITONITIS.

**Intestinal Incision and Drainage in Acute Peritonitis** has been recommended by Mixter[1] for the intestinal distension which is frequently seen in grave cases of general peritonitis, and which by causing paralysis of the intestine usually leads to a fatal result. In such cases the author believes it is safe to open the coils of intestine at as many points as may be necessary to thoroughly drain them. He recommends that they be drawn out of the wound, held over a basin, incised in from one to four places, and thoroughly emptied, after which they should be quickly washed off with hot saline solution, sewed up, and returned, and the abdominal incision closed. In some cases, particularly those that have had an abdominal incision on the right side, Mixter secures permanent drainage by introducing a tube into the most prominent part of the cæcum and retaining it as long as necessary. Through this tube salts and stimulants may be introduced if the stomach is not retentive.

**Peritonitis**, according to Cole,[2] is always a symptom demanding surgical consideration, but not necessarily operative interference. He concludes that (1) in localised cases of pelvic origin we should use saline cathartics and hot douches, followed later by such operative treatment (preferably by the vagina) as may be required; (2) in cases originating in the vermiform appendix, early operative interference offers the best chance in a considerable proportion of cases; (3) in circumscribed cases, of whatever origin, where the process has simply resulted in an abscess, it should be dealt with as such; (4) in a general septic peritonitis, of whatever origin, an operation alone gives any hope. Cases of recovery after operation for diffuse peritonitis are recorded by Holmes,[3] Kakels,[4] and Sheild.[5]

**Tubercular Peritonitis** is discussed by Thomson,[6] who does not regard the mere removal of the ascitic fluid as the essential element of success in laparotomy for these cases, because—(1) there is a comparative absence of improvement in cases in which the fluid has been aspirated; (2) improvement has resulted from laparotomy in cases where there has been no fluid to evacuate; (3) in cases in which the surgeon operated with the object of evacuating fluid, and because of the fluid being circumscribed or encysted it was not possible to reach the accumulation, recovery has ensued, although the fluid was allowed to remain within the abdomen. He also regards it as highly improbable that the entrance of air or gas has any influence in the beneficial results so obtained. In operating for tuberculous ascites air does not enter the peritoneal cavity, unless the procedure is modified with this object in view, and yet the number of the cases benefited by simple incision already amounts to several hundreds. He also rejects the theories that the improvement is due to the escape of toxins and ptomaines, the entrance of antagonistic organisms, the exposure of the peritoneum to sunlight, the anæsthetic, or the modification of the state of pressure within the abdomen. The author thinks it does not seem unreasonable to infer that the incision itself is the one constant and essential factor in the curative influence exerted by laparotomy, and that the benefit is due to the surgical interference *per se*, and not to any particular method of interference, nor to any of its incidental accompaniments. Thomson refers to the experiments of Nannotti and Baciocchi,[7] who induced tubercular peritonitis in dogs and rabbits. Of those "left to themselves" all died except one dog, but of those subjected to laparotomy death was delayed in the case of the rabbits, and seven dogs completely recovered, while others improved. The changes observed in the process of recovery consisted in granular disintegration of the bacilli, phagocytosis by epitheloid cells, degeneration of the cells constituting the tuberculous tissue, penetration of the latter by new vessels and connective tissue, leading to its absorption and replacement by scar tissue; these processes being precisely the same as those observed when recovery takes place without laparotomy, and they express the opinion that the operative interference stimulates or increases the reparative changes by the mechanical influence which it exerts on the impressionable peritoneum.

Thomson thinks that cases of the adhesive type, where there is no fluid in the abdominal cavity, while less favourable than the cases in which fluid accumulation is the cheaf feature, are capable of being favourably influenced by laparotomy, provided there is no serious tuberculous lesion elsewhere in the body. Jordan[8] also thinks that the dry form of tubercular peritonitis may be recovered from after simple incision of the abdominal cavity, and quotes cases supporting this view. The statistics of Rörsch are quoted to show that complete recovery is obtained after operation in about 70 per cent. of cases. Lenhart,[9] in praising the results of laparotomy in these cases, quotes the figures given by Aldibert. Of the 52 cases in which laparotomy was performed in children, there were 45 recoveries and seven deaths; of these 45, nine had lived for more than one year, and two for more than two years. It is to be remembered, however, that both in adults and children, if the symptoms are not urgent, and the patient's strength is not failing, we should not interfere surgically, as many of these cases recover spontaneously.

[1] Amer. Journ. of the Med. Sci., Nov., 1895, and Boston Med. and Surg. Journ., No. 9, 1895. [2] New York Med. Journ., June 1, 1895. [3] Brit. Med. Journ., July 6, 1895. [4] New York Med. Journ., July 6, 1895. [5] Lancet, Sep. 28, 1895, p. 789. [6] Practitioner, Nov., 1895, p. 440. [7] Centralblatt für Chir., No. 21, 1895. [8] Med. Week, Nov. 7, 1895, and Beit. Z. Klin. Chir., XIII., 3. [9] N. Y., Med. Rec., Oct. 26, 1895.

